# The Effects of Crocin on Bone and Cartilage Diseases

**DOI:** 10.3389/fphar.2021.830331

**Published:** 2022-01-19

**Authors:** Shayan Vafaei, Xuming Wu, Jiajie Tu, Seyed Noureddin Nematollahi-mahani

**Affiliations:** ^1^ Department of Anatomical Science, School of Medicine, Kerman University of Medical Sciences, Kerman, Iran; ^2^ Key Laboratory of Anti-Inflammatory and Immune Medicine, Anhui Collaborative Innovation Center of Anti-Inflammatory and Immune Medicine, Institute of Clinical Pharmacology, Anhui Medical University, Ministry of Education, Hefei, China

**Keywords:** crocin, bone, cartilage, inflammation, cell differentiation

## Abstract

Crocin, the main biologically active carotenoid of saffron, generally is derived from the dried trifid stigma of *Crocus sativus* L. Many studies have demonstrated that crocin has several therapeutic effects on biological systems through its anti-oxidant and anti-inflammatory properties. The wide range of crocin activities is believed to be because of its ability to anchor to many proteins, triggering some cellular pathways responsible for cell proliferation and differentiation. It also has therapeutic potentials in arthritis, osteoarthritis, rheumatoid arthritis, and articular pain probably due to its anti-inflammatory properties. Anti-apoptotic effects, as well as osteoclast inhibition effects of crocin, have suggested it as a natural substance to treat osteoporosis and degenerative disease of bone and cartilage. Different mechanisms underlying crocin effects on bone and cartilage repair have been investigated, but remain to be fully elucidated. The present review aims to undertake current knowledge on the effects of crocin on bone and cartilage degenerative diseases with an emphasis on its proliferative and differentiative properties in mesenchymal stem cells.

## Introduction

**FIGURE 1:**
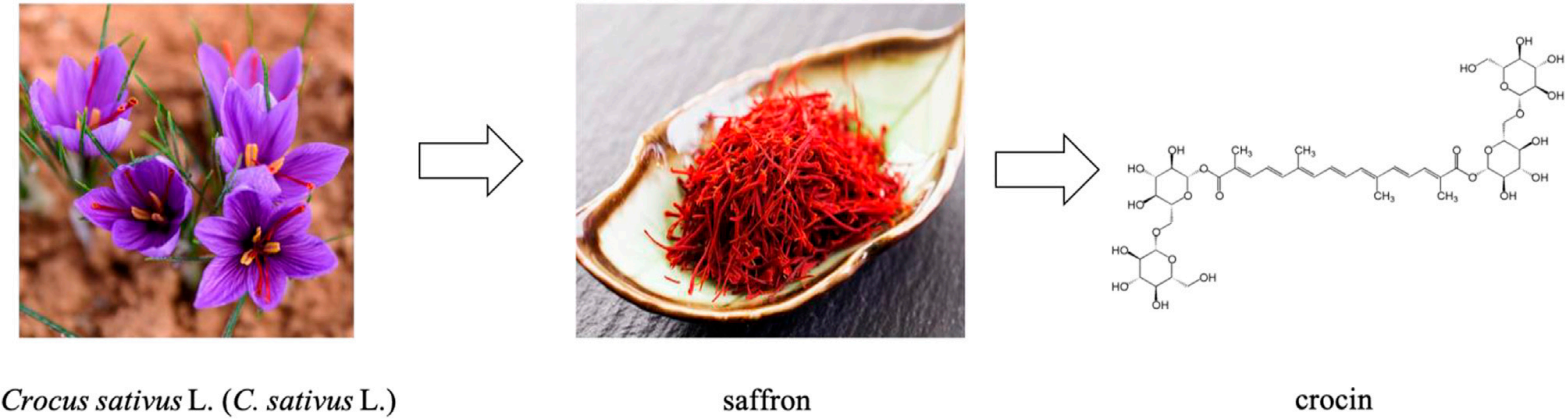
The picture of *C. Sativus L.*, saffron and crocin (molecular structure).

*Crocus sativus* L. (*C. sativus* L.) is one of about 88 species from the *Crocus* genus, which is part of the Iridaceae family. It is well known in herbal medicine and has attracted the attention of researchers because of its properties, especially its anti-inflammatory and proliferative capacities in bone and cartilage destructive diseases (Ríos et al., 1996; Mollazadeh et al., 2015). This plant is mainly cultivated in Iran, China, India, Azerbaijan, Turkey, Morocco, Greece, Spain, Italy, Mexico, and other places (Xue, 1982; Alavizadeh and Hosseinzadeh, 2014). It is a perennial herb that grows up to about 20 cm and usually produces 2-3 blue-purple flowers (Melnyk et al., 2010). The dried stigma, called saffron, is the most widely used part (Gismondi et al., 2012; Winterhalter and Straubinger, 2000). Because of the distinguished color, odor, and flavor, it is used as a food coloring and flavoring substances (Winterhalter and Straubinger, 2000; Caballero-Ortega et al., 2007; Mollazadeh et al., 2015). Carotenoids, the main metabolites of saffron, are responsible for the red color, smell, and bitterness (Srivastava et al., 2010; Gismondi et al., 2012). Water-soluble carotenoids can affect certain cellular pathways and molecules because of their ability to bind to a wide range of proteins, including membrane proteins, transcription factors, mitochondrial proteins, structural proteins, and enzymes (Hosseinzadeh et al., 2014; Li S et al., 2017). Among these biologically active components, there are four well-established ingredients that are likely responsible for the therapeutic potential of saffron, including crocin, crocetin, safranal, and picrocrocin (Pfander and Schurtenberger, 1982; Tsimidou and Tsatsaroni, 1993; Liakopoulou-Kyriakides and Kyriakides, 2002; Srivastava et al., 2010; Gohari et al., 2013; Hosseinzadeh and Nassiri-Asl, 2013). Crocin has five proper subsets; the principal one in saffron is α-crocin (Alonso et al., 2001; Ordoudi et al., 2015). Chemical studies have shown that crocin is a diester composed of the disaccharide gentiobiose and the dicarboxylic acid crocetin (Figure 1) (Alavizadeh and Hosseinzadeh, 2014). In the past, saffron was used as a sexual stimulant, and as a treatment for infertility and impotence (Asadi et al., 2014). Recent studies have revealed other therapeutic and pharmacological activities of saffron, such as neuroprotective (Baghishani et al., 2018; Haeri et al., 2019), neurogenetic (Ebrahimi et al., 2021), antidepressant (Shafiee et al., 2018), anti-apoptotic (Vafaei et al., 2020), antioxidant (Altinoz et al., 2016; Hatziagapiou et al., 2019), and anti-inflammatory (Nam et al., 2010; Lv et al., 2016) effects. Crocin is one of essential ingredients that responsible for the therapeutic effects of saffron. Specifically, the antioxidative properties of crocin involve several signaling pathways and molecules. For example, it modulates GPx, GST, CAT, and SOD (Korani et al., 2019), inhibits reactive oxygen species (ROS) and interacts with peroxidase (Mostafavinia et al., 2016). Overall, it inhibits free radicals (Ebadi, 2006) and affects certain pathways, such as CREB signaling (Zheng et al., 2007). Crocin also has anti-inflammatory properties via the downregulation of inflammatory marker levels such as interleukin (IL)-1ß, IL-6, tumor necrosis factor (TNF)-α, and insulin-like growth factor (IGF)-1, or through modulation of signaling pathways such as PI3K/Akt and Nuclear factor-kappa B (NF-κB) (Deng et al., 2018; Xie et al., 2019). It has been shown that crocin can polarize macrophages to the M2 (anti-inflammatory) phenotype by suppressing the p38 and JNK pathways. Therefore, its anti-inflammatory effects are associated with this pathway, in addition to other pathways (Zhu et al., 2019). Furthermore, crocin is metabolized in the liver and exerts protective effects on liver toxicity induced by morphine (Salahshoor et al., 2016) and nicotine (Jalili et al., 2015).

## Methods

Crocin exerts its effects under various conditions, and its antioxidant and anti-inflammatory properties contribute to the treatment of various diseases, including bone and cartilage inflammation. In this review, we summarized studies published through 2021 on the effect of crocin on bone and cartilage diseases. We chose crocin, bone, cartilage, and inflammation as keywords. The related articles were collected from online literature resources such as Web of Knowledge, PubMed, Scopus, and Google Scholar.

## Role of Crocin in Bone and Cartilage Diseases

### Crocin and Osteoarthritis

One of the most common joint diseases worldwide is osteoarthritis (OA), which is considered the main cause of disability in elderly people and often presents with pain and limited movement (Krasnokutsky et al., 2007; Silverwood et al., 2015; Dubin, 2016). OA severity varies from localized to chronic inflammation (Feldmann, 2001) and leads to joint cartilage degeneration, synovitis, and even bone remodeling (Benazzo et al., 2016). OA is reinforced by several factors such as obesity, age, trauma, mechanical stress, oxidative stress, and inflammation (Chen D. et al., 2017; Lim et al., 2017; Min et al., 2018; Yoo et al., 2018). In mild and severe OA, symptoms of inflammation are pronounced (Sinkov and Cymet, 2003), and inflammatory cytokines, including IL-2, interferon (IFN)-γ, TNF-α, and IL-1ß, are thought to be involved in the pathology (Goldring, 2000; Linton and Fazio, 2003; Chen D. et al., 2017). Overall, inflammatory cytokines lead to NF-κB signaling pathway activation, which can induce expression of matrix-degrading enzymes, such as matrix metalloproteinase (MMP) and C-reactive protein (CRP) 5, and increase erythrocyte sedimentation rate (ESR), which are involved in cartilage degeneration and osteoarthritis (Pennock et al., 2007; Sakkas and Platsoucas, 2007; Mohamadpour et al., 2013; Chen D. et al., 2017). MMPs, especially MMP1 and MMP3, destroy the extracellular matrix, thereby disrupting normal joint performance and leading to OA progression (Largo et al., 2003; Tardif et al., 2004; Burrage et al., 2006; Takaishi et al., 2008). Researchers have shown that the anti-inflammatory properties of crocin have a therapeutic effect on OA. In the study by Lei et al., OA rats were administered 30 mg/kg crocin daily for 10 days. After treatment, joint pain, IL-6 level, muscular lipid peroxidation (LPO), and Nrf2 levels were decreased, while citrate synthase (CS) activity, myosin heavy chain (MHC) IIα expression, glutathione production, and glutathione peroxidase activity were increased. They concluded that crocin could reduce OA symptoms by alleviating oxidative stress and inflammation and inhibiting JNK activity, which is an interesting property for OA treatment (Lei et al., 2017). A study by Ding et al. demonstrated the chondrogenic effects of crocin. In their study, crocin repressed IL-1ß expression and reduced the synthesis of MMP-1, -3, and -13 in chondrocytes, probably by blocking the NF-κB pathway. In the *in vivo* phase of their study, intra-articular injections of crocin were performed, and the results showed that crocin can reduce cartilage degeneration in OA-induced rabbit knees (Ding et al., 2013). In a study conducted by Li et al. on the anti-inflammatory effects of crocin on rat intervertebral discs, nucleus pulposus cells were isolated from rats and treated with different doses of crocin. Crocin reduced MMP-1, -3, and -13 overexpression, pro-inflammatory factors including IL-1β, TNF-α, IL-6, and inducible nitric oxide synthase (iNOS), and inhibited mitogen-activated protein kinase (MAPK) and JNK pathways (K. Li et al., 2015). In a clinical trial conducted by Poursamim et al., 40 patients with OA received Krocina (crocin tablets, 15 mg/daily) or placebo for 4 months. The results demonstrated that crocin reduced serum CRP and IL-17 levels. In addition, the number of regulatory T cells increased while the number of T helper and CD8^+^ cells decreased in crocin-and placebo-treated individuals, respectively. Finally, in the crocin group, the Treg/Th17 ratio shifted towards regulatory T cells (Poursamimi et al., 2020). The aforementioned reports demonstrate the possible curative potential of crocin on OA, which makes this herbal plant an appropriate candidate for OA treatment. In Table 1, a summary of studies on crocin and OA is presented.

**TABLE 1:** Brief summary of studies on crocin and OA.

Reference	Models/Crocin doses	Main results	Conclusion
Lei et al. (2017)	Rats/30 mg/kg daily for 10 days	Decrease in joint pain, IL-6 level, LPO, and Nrf2 expression; increase in CS activity, MHC IIα expression, glutathione production, and glutathione peroxidase activity	Crocin reduces OA symptoms by affecting oxidative stress, inflammation, and JNK activity
Ding et al. (2013)	chondrocyte culture, and 5–100 µM (50–1,000 mg/ml) intra-articular injection	Repression of IL-1ß, downregulation of mRNA and protein expression of MMP-1, -3 and -13	Crocin reduces inflammation *in-vitro* and regenerates rabbit knee cartilage
(K. Li et al., 2015 )	NP cells/10–100 µM (100–1,000 mg/ml)	Decrease MMP-1, -3, and -13 overexpression, IL-1β, TNF-α, IL-6, and iNOS, and inhibit MAPK and JNK pathways	Crocin reduces inflammation *in-vitro* and *ex-vivo*
Poursamimi et al. (2020)	OA patients/15 mg tabs/day for 4 months	Decrease CRP and IL-17, increase regulatory T cells, shifted Treg/Th17 ratio towards regulatory T cells	Crocin decreases inflammation in OA patients

### Crocin and Rheumatoid Arthritis

Rheumatoid arthritis (RA) is a chronic autoimmune disease characterized by synovitis and degeneration of the cartilage and underlying bone, which can lead to lasting joint disorders (Turco, 1963; Nakken et al., 2017; Dreher et al., 2019). In this comprehensive disease, joint symptoms are most prevalent, which develop and progress through inflammation (Hamerman, 1966; CADTH Common Drug Reviews, 2015; Borthwick, 2016; Szekanecz et al., 2016). Studies have demonstrated a pivotal role for inflammatory cytokines, including TNF-α, IL-1β, and IL-6, in RA initiation and progression (Benucci et al., 2012; do Prado et al., 2016; Duesterdieck-Zellmer et al., 2012; Furman et al., 2014; Giacomelli et al., 2016; Hreggvidsdottir et al., 2014; G.; Li et al., 2016). Similar to the above studies, there are some reports demonstrating the probable roles of oxidative stress in RA development (Kacsur et al., 2002; Meki et al., 2009; Filaire and Toumi, 2012; Radhakrishnan et al., 2014). Studies have also shown that some signaling pathways can affect the progression and prognosis of RA, including Wnt/β-catenin signaling pathways. The Wnt/β-catenin pathway can regulate inflammatory cytokine secretion, which can affect fibroblast-like synoviocyte (FLS) proliferation and give rise to bone metabolism/destruction (Brunt et al., 2018; Liang et al., 2019; Macedo et al., 2019; Wang et al., 2020; Miao et al., 2021). When the Wnt signaling pathway is activated, pro-inflammatory cytokines, including TNF-α and IL-1β*,* are produced (Wu et al., 2017; Brunt and Scholpp, 2018; Yuan et al., 2018). NF-κB, which acts as an RA initiator, is another important molecule involved in RA pathogenesis (Gilston et al., 1997; Makarov, 2001). It has been suggested that NF-κB activation occurs prior to type II collagen-induced arthritis (CIA), which is associated with autoimmunity to type II collagen, B cells, and T cells, especially Th17, macrophages, and cytokines (Mulherin et al., 1996; Ehinger et al., 2001; Murphy et al., 2003; Roman-Blas and Jimenez, 2006; Zhu et al., 2010; Hu et al., 2013; Al-Zifzaf et al., 2015). Given the inflammatory nature of RA initiation, and the anti-inflammatory effects of crocin, studies have been designed to understand the possible effects of crocin on RA inhibition and treatment. In a study by Hemshekhar et al., in 2012, 10–20 mg/kg crocin was administered for 15 consecutive days in a rat model of arthritis. They demonstrated that crocin modulates the serum levels of enzymatic and non-enzymatic inflammatory cytokines, including MMP-13, MMP-3, MMP-9, HAases, TNF-α, IL-1β, NF-κB, IL-6, COX-2, and PGE2, as well as ROS mediators, which were increased in the RA-induced rats. Furthermore, crocin also increased the levels of GSH, SOD, CAT, and GST. In addition, inhibiting the exoglycosidases cathepsin-D and tartrate-resistant acid phosphatase in the bones adjacent to the joints by crocin protected bone resorption (Hemshekhar et al., 2012). Rathore et al. administered three doses of crocin (25, 50, and 100 mg/kg) for 47 days in a mouse model of RA. They observed a reduction in TNF-α and IL-1β levels and an increase in SOD and GR activity when higher doses were administered (Rathore et al., 2015). Hu et al. injected 160 mg/kg crocin for 14 days into RA-induced rats. Paw swelling and ankle diameters in crocin-treated rats were significantly decreased as compared to controls. Histological analysis also showed that inflammation was reduced in the joints and other organs, such as the spleen. In addition, TNF-α and TGF-β1 levels decreased in synovial tissues (Hu et al., 2019). In a similar study, Liu et al. showed that the anti-inflammatory and anti-arthritic effects of 40 mg/kg crocin lasted for 15 days. Their study showed that MMP-1, -3, and -13 protein expression levels were decreased in RA-induced rats (Liu et al., 2018). At the same time, Li et al. showed similar results, in addition to a reduction in iNOS production. This study, along with others, showed that crocin has positive effects on RA-induced rats (Li X et al., 2017). In an *in vitro* study, Li et al. demonstrated that 500 µM (5,000 mg/ml) of crocin reduced the levels of TNF-α, IL-1β, and IL-6 in human FLS. In addition, crocin caused lower levels of *p*-IκBα, *p*-IκB kinase α/β, and p65 expression, demonstrating its effect on the NF-κB pathway. The *in vivo* phase of their study showed that crocin can decrease TNF-α, IL-1β, and IL-6 serum levels, and that NF-κB signaling could suppress inflammation in FLS in RA-induced mice (Li L et al., 2018). Wang et al. showed that crocin inhibits Wnt/β-catenin and the Wnt signaling pathway to reduce pain-related cytokines, and glial activation may reduce neuropathic pain in RA-induced rats (J. F. Wang et al., 2020). Collectively, crocin may be an efficient treatment for RA and is effective for its associated secondary complications. Table 2 summarizes these studies.

**TABLE 2:** Brief summary of studies on crocin and RA.

Reference	Models/Crocin doses	Main results	Conclusion
Hemshekhar et al. (2012)	Rats/10–20 mg/kg daily for 15 days	Decreased MMP-13, MMP-3, MMP-9, HAases, TNF-a, IL-1b, NF-κB, IL-6, COX-2, PGE2 and ROS.	Reduced RA symptoms by regulating oxidative stress, inflammation, and the levels of exoglycosidases, cathepsin-D and tartrate-resistant acid phosphatase
		Impression GSH, SOD, CAT, and GST. Inhibited levels of the exoglycosidases cathepsin-D, and tartrate-resistant acid phosphatase	
Rathore et al. (2015)	Mice/25, 50 and 100 mg/kg for 47 days	Reduction in TNF-α and IL-1β levels, increase in SOD and GR activity in 50 and 100 mg/kg treatments	Reduced inflammation and oxidative stress in 50 and 100 mg/kg treatments
Hu et al. (2019)	Rats/160 mg/kg for 14 days	Decreased paw swelling and ankle diameters, joint, spleen, and thymus inflammation, and levels of TNF-α and TGF-β1	Reduced RA symptoms and complications by reducing inflammation
Liu et al. (2018)	Rats/40 mg/kg for 15 days	MMP-1, -3, and -13 protein expression levels were decreased and decreasing inflammatory cytokines similar to previous studies	Reduced RA by reducing inflammation
Li X et al. (2017)	Rats/6.25–25 mg/kg	Reduction in iNOS and decrease in inflammatory cytokines similar to previous studies	Crocin has positive effects on RA-induced rats
Li L et al. (2018)	Synoviocytes/500 µM (5,000 mg/ml)	Reduced TNF-α, IL-1β, IL-6, *p*-IκBα, *p*-IκB kinase α/β, and p65 expression	Crocin had anti-inflammatory and anti-arthritic effects *in-vitro* and *in-vivo* through NF-κB signaling
( Wang et al., 2020 )	Rats/50 and 100 mg/kg	Reduced pain-related cytokines and glial activation by affecting Wnt/β-catenin and the Wnt signaling pathway	Reduced neuropathic pain in RA-induced rats

### Crocin and Osteoporosis

Osteoporosis (OP) is a progressive systemic skeletal disorder characterized by a reduction in bone mass and deterioration of bone tissue, which occurs following an imbalance of bone formation/absorption, leading to bone fragility. The risk of bone fractures, morbidity, and mortality increases in OP, which increases treatment expenses as well (NHI, 2001; Todd and Robinson, 2003; Bliuc et al., 2009; Bawa, 2010). There are numerous factors that contribute to OP pathogenesis, including metabolic syndrome (MetS), which involves abnormal glucose metabolism, dyslipidemia, hypertension, and abdominal obesity (Zhou et al., 2013). In MetS, fat tissue secretes inflammatory factors and hyperglycemia results in an increase in glycation end products, which leads to a reduction in bone mineral density (BMD) (Yamaguchi, 2014). Due to the positive effects of crocin on hypertension, body fat balance, and MetS, along with its anti-inflammatory properties, crocin as a potential treatment for osteoporosis should receive more attention (Sheng et al., 2006; Imenshahidi et al., 2015; Shafiee et al., 2017). In a study by Algandaby, 5 and 10 mg/kg crocin was administered to a rat model of metabolic syndrome-induced osteoporosis. In the crocin treatment group, bone tissue was histologically protected against OP effects, bone formation markers including serum alkaline phosphatase and osteocalcin increased, and bone resorption markers, including tartrate-resistant acid phosphatase and collagen cross-linking carboxyterminal telopeptide, were inhibited. In addition, crocin reduced TNF-α and IL-6 serum levels and oxidative stress in the epiphyseal tissue of rats. These results demonstrated that crocin may protect against MetS-induced osteoporosis (Algandaby, 2019). Another cause of OP is hormone (including estrogen, testosterone, and parathyroid hormone) deficiency, which usually effects cancerous bone and can cause a reduction in BMD. OP is more common in women than in men, and women over 50 years of age are more vulnerable to causes of OP, likely because of estrogen deficiency in the postmenopausal period (Hunter and Sambrook, 2000; Marcus, 2002; Johnell and Kanis, 2006; Sugerman, 2014; Noh et al., 2020). Cao et al. studied the effects of 5–20 mg/kg/day of crocin for 12 weeks in ovariectomized rats. They demonstrated that crocin protected rats from reduced BMD in L4 vertebrae and femurs, and prevented deterioration of the trabecular microarchitecture in rats caused by ovariectomy. A significant reduction in skeletal remodeling, as evidenced by lower levels of bone turnover markers, was also observed. Oxidative stress factors in the serum or bone tissue returned to near-normal conditions. Collectively, these results demonstrated that crocin administration can prevent OP in rats (Cao et al., 2014). In an *in vitro* study by Nie et al., crocin was used to protect against glucocorticoid-induced osteoporosis and osteonecrosis by inhibiting the ROS/Ca^2+^-mediated mitochondrial pathway. They showed that crocin decreases mitochondrial transmembrane potential and increases ROS and intracellular Ca^2+^ levels following induction of OP by dexamethasone in osteoblasts. In addition, the expression levels of B-cell lymphoma-2(Bcl-2) and mitochondrial cytochrome c (Cyt-C) were upregulated, and cleaved caspase-9, cleaved caspase-3, Bcl-2-associated X protein, and cytoplasmic Cyt C were downregulated by crocin (Nie et al., 2019). Taken together, these studies demonstrated that crocin is a potential medicine for OP treatment. Table 3 shows the relationship between crocin and OP.

**TABLE 3:** Brief summary of studies on crocin and OP.

Reference	Models/Crocin doses	Main results	Conclusion
Algandaby, (2019)	Rats/5–10 mg/kg daily for 12 weeks orally	Protected from histological changes in bone, increased serum alkaline phosphatase and osteocalcin, decreased tartrate-resistant acid phosphatase and collagen cross-linking carboxyterminal telopeptide and TNF-α and IL-6 oxidative stress	Crocin may be effective against MetS-induced osteoporosis
Cao et al. (2014)	Rats/5–20 mg/kg daily for 12 weeks	Reduction in skeletal remodeling and oxidative stress factors, increase in BMD and trabecular microarchitecture	Administration of crocin for 14 weeks can prevent OP in rats
Nie et al. (2019)	MC3T3-E1 cell line/100 µM (1,000 mg/ml)	Upregulated expression levels of Bcl-2 and Cyt C, and downregulated caspase-9, caspase-3, Bcl-2-associated X protein, and cytoplasmic Cyt C, and increased levels of ROS and intracellular Ca2+	Crocin may have a therapeutic effect on dexamethasone-induced apoptosis of osteoblasts via inhibition of the ROS/Ca2+-mediated mitochondrial pathway *in-vitro*

### Effects of Crocin on Cell Differentiation

Bone regeneration is a complex procedure that occurs in abnormal conditions, such as bone degenerative diseases and fractures, but is insufficient and inefficient in some circumstances (Marzona and Pavolini, 2009; Dimitriou et al., 2011). Following the inflammatory phase of bone defects, there is a proliferative phase called the mesenchymal activation phase. During this phase, mesenchymal stem cells (MSCs) differentiate into chondrocytes and osteoblasts, which facilitate bone regeneration, either through endochondral ossification or intramembranous ossification (Knight and Hankenson, 2013). Bony tissue cells include osteoclasts, osteoblasts, and osteocytes, which are involved in bone regeneration and remodeling. These cells are derived from MSCs depending on the environmental stimulants that coordinate bone formation and bone absorption (Boyle et al., 2003; Zaminy et al., 2008; Knight and Hankenson, 2013; Noh et al., 2020). For example, studies have demonstrated that bone marrow MSCs (BMSCs) as multipotent stem cells can differentiate into bone and cartilage cells. This occurs through the expression of different growth factors, including platelet-derived growth factor (PDGF), bone morphogenetic proteins (BMPs), and transforming growth factor-β(TGF-β), and likely via the ERK and JNK MAPK signaling pathways. However, these growth factors are highly limited in these cells owing to rapid degradation and high cytotoxicity, as well as the high financial cost of these factors; thus, it is desirable to investigate novel osteoblastic inducers, especially natural products (Friedman et al., 2006; Fan et al., 2011; Mostafa et al., 2012; Yu et al., 2012; Udalamaththa et al., 2016; Li C et al., 2017). Baharara et al. (2014) reported successful differentiation of BMSCs into osteoblasts following treatment with crocin, which was confirmed by an increase in alkaline phosphatase (ALP) activity, cell mineralization, and osteocalcin gene expression (Baharara et al., 2014). Kalalinia et al. (2018) demonstrated that 12.5–50 µM (125–500 mg/ml) crocin is not cytotoxic based on the MTT assay and IC_50_ calculation. Moreover, at these concentrations, it may enhance osteogenesis in BMSCs, as measured by ALZ intensity, ALP activity, and ALP mRNA expression. Thus, crocin can be considered a safe substance to promote the osteogenic differentiation of BMSCs (Kalalinia et al., 2018). Li et al. (2017) also studied the osteogenic effect of crocin both *in vitro* and *in vivo*. For the *in vitro* study, they treated human BMSCs with crocin and demonstrated an increase in ALP activity and calcium nodule formation (assayed by alizarin red S staining). In addition, they treated male rats with femoral head osteonecrosis with crocin and showed considerable histopathological changes in the femoral head tissues with H&E staining. Western blotting and q-PCR assays showed an increase in the expression levels of RUNX2, COL1A1, and OCN, and a decrease in GSK-3β phosphorylation in both bone tissue and BMSCs after treatment with crocin, in a dose-dependent manner. These researchers suggested that crocin has potential for use in the treatment of osteogenic diseases in the future (B. Li et al., 2020). Koski et al. administered crocin over 7 weeks to human fetal osteoblasts and observed an increase in cell proliferation. In addition, crocin decreased human osteosarcoma (MG-63) cells viability *in vitro*. In contrast, the *in vivo* application of crocin showed pro-apoptotic and anti-inflammatory effects in a rat model of femoral inflammation. These results suggest that crocin may have a therapeutic effect on osteosarcoma regulation and potential for use in wound healing during bone tissue regeneration (Koski et al., 2020). Studies have shown that in some diseases involving bone degeneration and dysregulation of bone homeostasis besides osteogenesis, the influence of osteoclast formation and osteo-immunomodulation is important (Chen et al., 2017b; Chen et al., 2017c). On the other hand, M2 macrophages (anti-inflammatory macrophages) secrete cytokines such as BMP-2 that contribute to osteogenesis (Yuan et al., 2017). Note that crocin may be effective in macrophage polarization and promotion of the M2 phenotype (Li J et al., 2018). Zhu et al. showed that crocin promoted macrophage polarization toward the M2 phenotype and reduced the expression of anti-inflammatory cytokines *in vitro* and *in vivo*. In addition, pre-treatment of macrophages with crocin induced the osteogenic differentiation of BMSCs in co-culture media. This is probably due to the inhibition of p38 and c-Jun N-terminal kinase signaling. This study indicated that crocin has therapeutic potential for bone degenerative disease by inducing M2 macrophage polarization, which results in inflammation reduction and osteogenic differentiation of BMSCs (Zhu et al., 2019). The above-mentioned studies have emphasized that crocin may have a positive effect on osteogenesis by promoting osteoblastic differentiation. A summary of these studies is provided in Table 4.

**TABLE 4:** Studies on the effect of crocin on osteoblastic differentiation.

Reference	Models/Crocin doses	Main results	Conclusion
Baharara et al. (2014)	BMSCs/60–80 µM (600–800 mg/ml)	Increased alkaline phosphatase (ALP) activity, cell mineralization, and osteocalcin gene expression	crocin may have effect on osteoblastic differentiation of BMSCs
Kalalinia et al. (2018)	BMSCs/12.5–50 µM (125–500 mg/ml)	Increased ALZ intensity, ALP activity, and ALP mRNA expression, was not cytotoxic using MTT test and IC_50_ calculation	Crocin can be considered a safe substance to promote osteogenic differentiation of BMSCs
(B. Li et al., 2020 )	hBMSCs/10–50 µM (10–500 mg/ml)	Increased LAP activity, calcium nodules, and RUNX2, COL1A1, and OCN expression, decreased GSK- 3β phosphorylation	Crocin is effective in *in-vitro* and *in-vivo* osteogenic models
Zhu et al. (2019)	M2 macrophages and BMSCs/40 and 80 µM (400–800 mg/ml)	Promoted M2 phenotype that was decreased in anti-inflammatory cytokine-induced osteogenic differentiation of BMSCs in co-culture with pre-treated macrophages through inhibition of p38 and c-Jun N-terminal kinase signaling	Crocin has therapeutic potential for bone degenerative diseases through induction of M2 macrophage polarization, resulting in inflammation reduction and osteogenic differentiation of BMSCs
Koski et al. (2020)	hFOBs and MG-63 cell line, Rats/45 µg (450 mg/ml)	Increased osteoblast proliferation and decreased osteosarcoma viability and pro-apoptotic and anti-inflammatory effects *in-vivo*	Crocin has a potential therapeutic effect on osteosarcoma regulation and uses for wound healing during bone tissue regeneration

In some pathological conditions related to bone loss-associated diseases (osteoporosis, arthritis, osteomyelitis, etc.), osteoclast cells are activated, leading to bone resorption. Under similar conditions, an imbalance occurs between osteoblast activities (leading to bone formation) and osteoclast activities (leading to bone resorption) (Boyle et al., 2003; Walsh and Gravallese, 2010; Redlich and Smolen, 2012). Stimulation of hematopoietic stem cells (HSCs) by inflammatory cytokines, such as IL-1, IL-6, and TNF-α (which are inhibited by crocin, as mentioned above) or other factors such as monocyte/macrophage colony-stimulating factor (M-CSF) and activation of receptor activator of nuclear factor kappaB (RANK) with its ligand (RANKL) can lead to osteoclast differentiation (Udagawa et al., 1999; Azuma et al., 2000; Teitelbaum, 2000; Ross, 2006; Walsh and Gravallese, 2010; Redlich and Smolen, 2012; Yamashita et al., 2012; Xu and Teitelbaum, 2013; Yokota et al., 2014). Studies have indicated that RANKL, a membrane protein of the TNF family, plays a role in osteoclast differentiation (Yasuda et al., 1998; Takayanagi et al., 2000; Roodman, 2006). RANKL is expressed on osteoblast cell membranes in response to stimulatory factors and then engages RANK on osteoclast cell membranes, along with activation of the NF-κB and MAPK signaling pathways. The final product of these cascades is the expression of tartrate-resistant acid phosphatase (TRAP) and other enzymes, which are involved in osteoclast-mediated bone resorption (Asagiri and Takayanagi, 2007). Fu et al. demonstrated that crocin suppresses osteoclast differentiation and function by directly inhibiting RANKL in bone marrow-derived macrophages (BMM). Downregulation of the NF-κB pathway and reduction in osteoclast-specific gene expression, including NFATc1, c-Fos, and cathepsin, are involved, leading to inhibition of bone resorption activity (Fu et al., 2017). A similar study by Shi et al. demonstrated that crocin downregulates osteoclast differentiation via inhibition of JNK and NF-κB signaling pathways in BMM cells *in vitro*. In the crocin-treated group, osteoclast markers including NFATc1, c-Fos, and cathepsin K, were downregulated. An inhibitor of κBα degradation and NF-κB p65 subunit nuclear translocation was suppressed, while c-Jun N-terminal kinase (JNK) was activated, resulting in the inhibition of RANKL in BMM. These results demonstrated that crocin decreased osteoclastogenesis in BMM (Shi et al., 2018). Suh et al. showed that crocin treatment decreased gene expression of TRAF6, Akt2, ERK1, OSTM1, and MMP-9, which are related to osteoclast differentiation and function and bone resorption *in vitro*, as well as a reduction in bone resorption activity of osteoclasts (Suh et al., 2019). These studies demonstrate the potential therapeutic effect of crocin on osteoclast and bone resorption dysfunction, as well as bone loss-associated diseases. A summary of these studies is provided in Table 5.

**TABLE 5:** Studies on the effect of crocin on osteoclastic inhibition.

Reference	Models/Crocin doses	Main results	Conclusion
Fu et al. (2017)	BMMs/100 µM (1,000 mg/ml)	Inhibition of RANKL, downregulation of NF-κB pathway, and reduction of NFATc1, c-Fos and cathepsin levels	Crocin suppresses osteoclast differentiation and function and inhibits bone resorption activity
Shi et al. (2018)	BMMs/10–40 µM (100–400 mg/ml)	Downregulation of NFATc1, c-Fos and cathepsin K, inhibition of κBα degradation, NF-κB p65 subunit nuclear translocation suppression, and JNK activation resulted in inhibition of RANKL	Crocin downregulates osteoclast differentiation via inhibition of JNK and NF-κB signaling pathways and decreases osteoclastogenesis in BMMs
Suh et al. (2019)	RAW264.7 cell line/2–10 µM (20–100 mg/ml)	Decreased gene expression levels of TRAF6, Akt2, ERK1, OSTM1, and MMP-9	Crocin decreases osteoclast function and differentiation and bone resorption *in-vitro*, as well reduction in bone resorption activity of osteoclasts

## Conclusion

Studies have shown that crocin, the main biologically active component of saffron, has anti-inflammatory and antioxidant effects. In addition, crocin has potential therapeutic effects on bone and cartilage diseases that involve inflammation and accumulation of free radicals, including OA, RA, and osteoporosis. Crocin can reduce oxidative stress and inflammatory cytokines via inhibiting molecular pathways include Wnt, MAPK and JNK signaling pathway. It modulates PI3K/Akt and NF-κB signaling pathways and polarizes macrophages to the M2 (anti-inflammatory) phenotype by suppressing the p38 and JNK pathways. Crocin also has proliferative and anti-apoptotic effects, especially on osteoblasts, and positive effects on osteoblastic differentiation of MSCs, while it also inhibits osteoclast activity. These data suggest promising potential therapeutic use of crocin in bone degenerative and bone-loss diseases, which require more precise laboratories and clinical trials. For example, crocin in high doses exhibited a cytotoxic effect and acts as an apoptotic promotor (Li et al., 2013), so it seems that further researches are needed to the determination of proper crocin dosage for both *in-vitro* and *in-vivo* studies. Also, the molecular mechanisms of various crocin effects are not recognized very well yet, so future studies may need to clarify the molecular mechanisms that they are involved. Overall regarding the beneficial effects of crocin in bone and cartilage diseases and due to lack of human studies in crocin effects in this field, the need for human trials is felt and future studies can be done in this research area.
